# New Insights into the Pivotal Role of the Amygdala in Inflammation-Related Depression and Anxiety Disorder

**DOI:** 10.3390/ijms231911076

**Published:** 2022-09-21

**Authors:** Ping Hu, Ying Lu, Bing-Xing Pan, Wen-Hua Zhang

**Affiliations:** 1 Institute of Translational Medicine, Nanchang University, Nanchang 330001, China; 2 Department of Biological Science, School of Life Science, Nanchang University, Nanchang 330031, China; 3 Laboratory of Fear and Anxiety Disorders, Institutes of Life Science, Nanchang University, Nanchang 330031, China

**Keywords:** depression, anxiety disorder, inflammation, amygdala

## Abstract

Depression and anxiety disorders are the two most prevalent psychiatric diseases that affect hundreds of millions of individuals worldwide. Understanding the etiology and related mechanisms is of great importance and might yield new therapeutic strategies to treat these diseases effectively. During the past decades, a growing number of studies have pointed out the importance of the stress-induced inflammatory response in the amygdala, a kernel region for processing emotional stimuli, as a potentially critical contributor to the pathophysiology of depression and anxiety disorders. In this review, we first summarized the recent progress from both animal and human studies toward understanding the causal link between stress-induced inflammation and depression and anxiety disorders, with particular emphasis on findings showing the effect of inflammation on the functional changes in neurons in the amygdala, at levels ranging from molecular signaling, cellular function, synaptic plasticity, and the neural circuit to behavior, as well as their contributions to the pathology of inflammation-related depression and anxiety disorders. Finally, we concluded by discussing some of the difficulties surrounding the current research and propose some issues worth future study in this field.

## 1. Introduction

Depression and anxiety disorder are the most common mental diseases affecting hundreds of millions of people worldwide [1]. The comorbidity rate of anxiety disorder and depression is very high, with 74% of depressed patients having anxiety symptoms, while 61% of anxious patients have depression symptoms [2]. Stress exposure is widely accepted as a critical contributing factor to psychological and neuropathological disorders [3]. Especially during the COVID-19 pandemic, the high pressure of increasingly demanding work and life has led to a sharp rise in the incidence of mental diseases [4]. Dysfunction of monoaminergic neurotransmission (monoamine hypothesis) by stress has long been known as the most prevalent hypothesis in the pathophysiology of depression [5,6]. First-line antidepressant therapy based on the monoamine hypothesis has prevailed for nearly 70 years, which aims to improve the level of monoamines (such as serotonin and norepinephrine) in the brain [6]. However, the most severe problem of monoamine-related therapy is that the therapeutic effect is limited since it usually takes several weeks or months to reach beneficial effects and up to 30% of patients do not respond to typical antidepressant medications [6,7], suggesting that the monoamine hypothesis may not fully represent the pathophysiological mechanism of depression. In this case, novel mechanisms are needed to clarify the pathogenesis of these mood disorders, which may help to develop new therapeutic drugs and strategies.

In recent years, many studies have consistently shown that stress-induced neuroinflammation and subsequent changes in the brain regions involved in emotion regulation play an essential role in the pathological development of neuropsychiatric diseases including depression and anxiety disorders [8,9,10,11]. The evidence includes: (1) the administration of inflammatory inducers such as lipopolysaccharide (LPS), interleukin-1β (IL-1β), TNF-α, or other cytokines can induce depression-like behavior in rodents [12]; (2) levels of inflammatory markers such as, IL-1β, IL-6, C-reactive protein (CRP), toll-like receptor 3 (TLR3), and TLR4 were increased in patients with depression and anxiety [13]; (3) the prevalence of depression in people with autoimmune diseases is much higher [14,15], with many patients with chronic inflammatory autoimmune disease, such as rheumatoid arthritis [16], multiple sclerosis [17], and stroke [18], often exhibiting depressive symptoms; and (4) patients who experienced cytokine treatment often have the characteristics of depression. Interferon-alpha (IFN-α) immunotherapy can cause wide-range psychiatric side effects, including anorexia, insomnia, anxiety, and other depressive symptoms [19,20], while anti-inflammatory therapy results in remission of depression [21]. These findings from rodents and humans have consistently proved that inflammation and cellular immune activation are the critical factors of depression, laying the foundation for the theory of the inflammatory response to depression.

Accumulating evidence from the past decades has shown that inflammation induces psychological and neuropathological disorders by influencing neuronal excitability, neurotransmitter release, receptor, and transporter expression through peripheral hormones and autonomic responses [22,23]. A number of animal and human studies have revealed that the amygdala, ventral hippocampus, and medial prefrontal cortex (mPFC) are extensively involved in the occurrence of anxiety, depression, and related behavioral regulation [24,25]. Among them, the amygdala, one of the kernel brain regions mediating stress-coping located in the deep temporal lobe, is considered the hub center for processing emotionally salient stimuli and implementing appropriate behavioral responses [26,27]. Studies from multiple animal models have suggested that the amygdala is overactivated in response to stress [28,29]. Human neuroimaging data have also shown that amygdala activity increases in patients with stress or mood disorders [30,31]. In recent years, interest has been attracted to revealing the mechanisms underlying inflammation in the amygdala. However, relatively few reviews have focused on summarizing the effects of the stress-induced inflammatory response on amygdala function and their contribution to the development of anxiety disorder and depression.

In this review, we first summarized the mechanisms of stress-induced inflammation (peripheral inflammation and central inflammation) and the crosstalk between peripheral inflammation and neuroinflammation in the brain. Then, the effects of inflammation on the functional changes in the amygdala and the relationship between inflammation and stress-related depression and anxiety disorders were systematically discussed from the perspectives of molecular signaling, cellular function and neural plasticity, circuit and behavioral output. Finally, we addressed some of the current issues and thoughts surrounding the amygdala research on the mechanism of inflammation-induced depression and anxiety disorders and proposed some research questions worthy of future research in this field.

## 2. Stress and Inflammatory Response

All living organisms strive to reach and maintain a dynamic equilibrium state called homeostasis, which is threatened by real or perceived physical, emotional, or psychological events, known as “stressors” [3,32]. From a broad perspective, stressors can be divided into psychological and systemic stressors. Psychological stressors are often defined as an imagined or existing social threat, e.g., aversive environmental stimuli, predator-related cues, and failure to meet internal drives, while systemic stressors mainly refer to actual disturbances to physiological states, such as bleeding, infection, and pain [33]. Notably, the stress-coping strategies vary depending on the intensity, duration, and timing of the stressors. Acute mild stress promotes the variability and adaptation that allow the organism to cope well with real or perceived threats that are critical for survival. In contrast, severe and prolonged stress (chronic stress) causes various maladaptive stress responses and leads to multiple psychiatric diseases, including depression, anxiety disorders, and post-traumatic stress disorder [34,35].

In recent years, numerous studies have indicated that the stress-induced activation of macrophages and microglia, which in turn leads to aberrant peripheral and central inflammation, plays a pivotal in the pathogenesis of mental diseases. Most noteworthy, inflammation can occur throughout the lifespan, making it a high risk for depression and anxiety disorders. For example, a recent study showed that exposing mice to LPS during early developmental stages increases the capacity of microglial engulfment, which induces long-lasting neuronal maladaptation, thus promoting the development of depression-like symptoms during adolescence [36].

### 2.1. Stress and Peripheral Inflammatory Response

**Figure 1:**
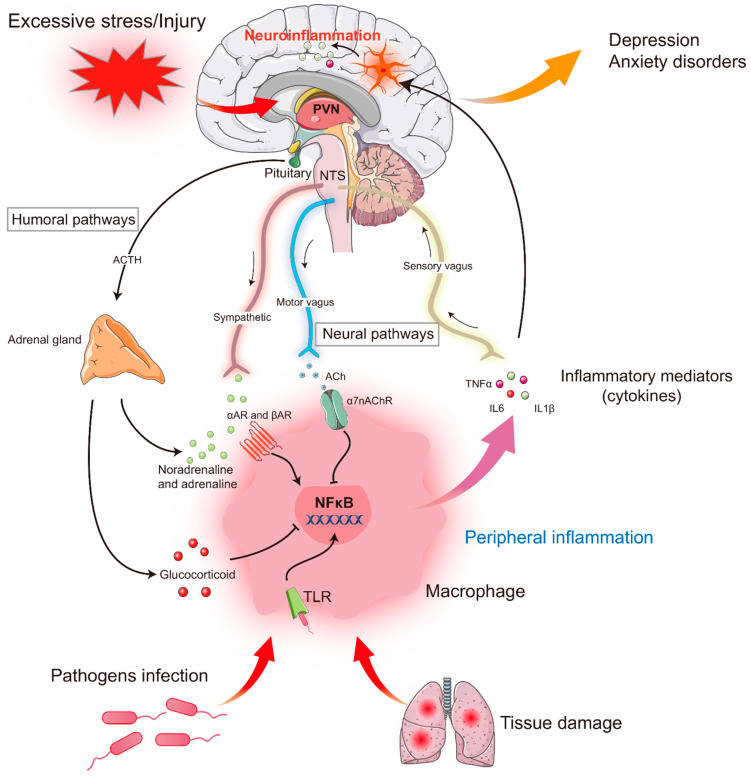
Stress-induced periphery inflammation and neuroinflammation in stress-related disorders: mechanisms and consequences interaction between the immune system, HPA axis, and sympathetic nervous system. Exposure to traumatic and stressful events in individuals may facilitate increased immune activity in both the periphery and the central nervous system (CNS) by activating the HPA axis and the sympathetic nervous system (SNS). HPA axis activation results in the release of glucocorticoids, which modulate the inflammatory response by suppressing the expression of pro-inflammatory cytokines by immune cells. However, overactivity of the SNS increases the release of pro-inflammatory cytokines. These cytokines access the brain via afferent fibers (e.g., vagus nerve) or through the damaged blood–brain barrier to activate microglia, which in turn contribute to neuroinflammation via secretion of pro-inflammatory cytokines in the brain.

During injury, disease, or infection by bacteria and viruses, the immune system is activated, and plenty of pro-inflammatory cytokines are produced and released by immune cells such as macrophages and T and B lymphocytes, which act as mediators of the innate immune response. Notably, a growing body of studies have shown that psychological or emotional stress can also trigger the activation of the peripheral immune system, leading to the release of multiple pro-inflammatory cytokines [37] (Figure 1). Psychological stress might alter immune function via the direct innervation of lymphatic tissue through the rapid activation of the hypothalamic–pituitary–adrenal (HPA) axis and the sympathetic–adrenal–medullary (SAM) system, two major components involved in stress coping [33,37,38]. In response to psychological stress, the hypothalamus secretes corticotropin-releasing hormone (CRH) to initiate a stress response. Higher levels of CRH cause the pituitary to release the adrenocorticotropic hormone, which subsequently triggers the synthesis and release of glucocorticoids (corticosterone in rodents, cortisol in humans) in the adrenal glands. In addition, neurons in the paraventricular nucleus of the hypothalamus project heavily to the locus coeruleus (LC), also known as the norepinephrine centers in the brainstem. The LC sends direct projections to the autonomic nervous system in the spinal cord, including sympathetic and parasympathetic preganglionic neurons, which increase sympathetic activity and decrease parasympathetic activity by activating adrenergic and muscarinic receptors [39]. The sympathetic nervous system is activated and stimulates adrenal medulla cells to release epinephrine and norepinephrine into the blood, which modulates the release of cytokines such as TNF-α and IL-6 by acting on α- and β-adrenergic receptors on immune cells (macrophages) and induces peripheral immune responses [40]. The activation of the afferent vagus nerve conveys peripheral inflammatory signals to the brain and then activates the anti-inflammatory efferent vagus nerve to suppress pro-inflammatory cytokine release and inhibit inflammation, known as “the cholinergic anti-inflammatory pathway” [41,42,43].

It should be noted that stress is common throughout life. The level of stress-induced inflammation and its influence on emotional behavior are closely related to stress intensity. In the face of acute stress, the body’s immune system will respond quickly in a short time by releasing inflammatory factors in response to stress. However, pro-inflammatory cytokines usually disappear after completing their task; when the stress persists (chronic stress), these cytokines continue to increase. Then, the persistently existing cytokines begin to have harmful effects on the body and induce changes in the brain regions related to emotion regulation, which subsequently cause mental illnesses such as depression and anxiety disorders.

### 2.2. Stress and Central Inflammatory Response

Unlike peripheral inflammation responses mediated by macrophages, central inflammation is mainly caused by microglia activation. Microglia are resident macrophages of the central nervous system (CNS), which constitute 5–10% of total brain cells and are the brain’s first line of defense against trauma, pathogens, infection, or brain diseases, a function similar to peripheral macrophages [44,45]. Microglia have high morphological plasticity. In the physiological state, microglia are highly branched in the resting state with a short protrusion, which can monitor the surrounding microenvironment. After brain injuries such as trauma, inflammation, infection, or disease, microglia can be rapidly activated and undergo morphological changes such as cell body enlargement and synaptic shortening, secrete a variety of pro-inflammatory cytokines, and activate phagocytosis [46,47]. It is widely accepted that activated microglia exert dual functional states, namely the classical activation state, which produces a large number of pro-inflammatory cytokines such as TNFα, IL-1β, IL-6, reactive oxygen species, and nitric oxide, thus exacerbating the neuroinflammatory state, and an alternative activation state, which is associated with an anti-inflammatory function. Notably, appropriate cytokines produced by microglia in the brain are critical positive regulators of several CNS functions, such as maintaining neuroplasticity [48,49]; however, the excessive or prolonged activity of inflammatory cytokines disrupts multiple neuronal functions, including impaired neurotransmitter signaling and the blockage of neurotransmitter synthesis, reuptake, and release [50]. This, in turn, results in the dysfunction of the neural circuits involving emotion and cognition [51].

Accumulating evidence from animal studies has revealed that restraint stress, unavoidable footshock stress, and chronic unpredictable stress can lead to the aggregation and activation of microglia in emotion-related brain regions such as the amygdala, prefrontal cortex, and hippocampus in rodents [52,53,54]. On the other hand, in patients with severe depression, microglia activation was observed in the cerebral cortex, prefrontal cortex, and the anterior cingulate cortex as well as the insula cortex, and the activation of microglia in the insula cortex was highly correlated with the severity of depression [55,56]. Studies have identified several possible ways that participate in the activation of microglia induced by stress. First, stress activates the HPA axis to release a large number of glucocorticoids, which results in the activation of microglia due to the abundant glucocorticoid receptors on the cell surface [57]. Second, stress promotes the expression of pro-inflammatory cytokines such as IL-1β in the CNS through the release of sympathetic norepinephrine and the activation of β-adrenergic receptors, thus leading to the initiation of microglial activation [58,59,60].

## 3. From the Peripheral to the Central Inflammatory Response

**Figure 2:**
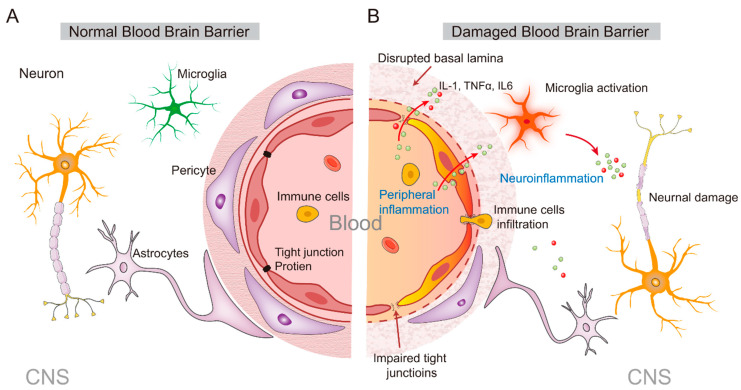
Increased periphery inflammation leads to disruption of the blood–brain barrier (BBB). (**A**) In healthy BBB, tight junctions between brain endothelial cells form the primary physical barrier that prevents the entry of large and potentially toxic molecules into the brain. Endothelial cells are encompassed by basal lamina, pericytes, and astrocytic endfeet. Pericytes and astrocytic endfeet interact closely with the endothelial cells and can help maintain BBB integrity. (**B**) Inflammation, caused by an infection or virus, can lead to immune cell infiltration. Increased periphery inflammation has a detrimental effect on the integrity of the BBB at various levels, including endothelial cell degradation or shrinkage, altered paracellular transport pathways via loss of tight junction proteins, as well as dysfunction of pericytes and astrocytes. Loss of BBB integrity makes it more permeable, allowing immune cells and inflammatory cytokines to enter the brain parenchyma, which in turn leads to microglial activation to induce neuroinflammation. Neurons may experience demyelination or become damaged.

Numerous animal studies have shown that peripheral inflammatory stimuli can activate microglia cells in the brain [61]. Due to the blood–brain barrier (BBB), the peripheral and central immune systems are relatively independent. However, under the continuous influence of chronic stress, the function of the immune system is downregulated, and peripheral inflammatory cells and inflammatory mediators can penetrate the CNS. Subsequently, microglia and astrocytes are activated to cause secondary neuroinflammation by producing pro-inflammatory cytokines such as TNF-α, IL-1β, and IL-6 (Figure 2).

Notably, cytokines cannot be passively diffused across the BBB because of their high molecular weight and hydrophilic properties, but they can enter the brain parenchyma through the humoral route (Figure 2). These processes begin in the circumventricular organs or choroid plexus and subsequently diffuse to other brain regions [62]. In the circumventricular organs, IL-1β increases prostaglandin 2 expression, which acts as a second messenger to transmit signals into the brain [63]. In addition, cytokines such as IL-1β, IL-1α, IL-6, and TNFα, have been found to enter the brain through an active transport mechanism mediated by saturable transporters [51]. In addition, cytokines can also activate glial cells in circumventricular organs, meninges, and choroid plexus to produce cytokines such as IL-1β [14,62].

In addition to the humoral routes, cytokines can also target the autonomic nerve afferents that innervate the infection site and transmit signals into the brain through the vagus nerve [64] (Figure 2). The vagus nerve, which includes sensory neurons that innervate most of the internal organs, can express IL-1 receptors [65], and can be stimulated by local cytokines to relay peripheral inflammatory signals to relevant brain regions such as the nucleus of the solitary tract (NTS) and lateral medulla [66]. On the other hand, activation of the vagus nerve can further activate the parabrachial nucleus and limbic brain regions through its projection [51], thereby increasing cytokine levels in the brain. For example, intraperitoneal injection of bacteria-derived lipopolysaccharides (LPS) triggers a robust inflammatory response and leads to a rapid increase in the expression of c-Fos (a biomarker of neuronal activity) in the NTS [67]. Once the peripheral cytokine signals enter the brain, they can activate their receptors and trigger an inflammatory cascade, thus amplifying the peripheral inflammatory signal and exerting the central effect of cytokines by affecting neuroimmunity, neuroendocrine, and behavior.

Clinical and animal studies have consistently shown that peripheral inflammation can disrupt the normal function of the BBB through multiple pathways, leading to secondary inflammatory responses in the CNS and the developing of neurological diseases [68]. The BBB is composed of vascular endothelial cells with tight junctions, which limit the body’s peripheral inflammatory cytokines, inflammatory cells, metabolites, neurotoxic substances, and pathogens in the blood from entering the CNS [68,69]. However, in the case of brain diseases such as infection and neurodegenerative diseases, the BBB can be destroyed, and then peripheral immune cells can enter the brain to induce a central inflammatory response, which further aggravates the BBB damage [68]. With the help of chemokines, peripheral immune cells, including macrophages, lymphocytes, and natural killer cells, could be recruited to the central inflammatory site, thus aggravating the central inflammatory response. There is evidence that BBB damage caused by inflammation is correlated to the integrity of tight junctions (Figure 2). Studies in elderly mice found that peripheral inflammation induced by LPS challenge can degrade the occludin and caludin-5, two kinds of tight junction proteins, which subsequently increases BBB permeability and affects its normal function [70,71]. On the other hand, the damage of cerebrovascular endothelial cells by peripheral inflammation is another important cause of BBB damage. For example, it has been reported that LPS inhibited p-glycoprotein activity and induced the secretion of active matrix metalloproteinases, which disrupt the function of vascular endothelial cells such as leading to endothelial cell apoptosis or the destruction of the ultra-microstructure, resulting in abnormal BBB function [71,72]. In addition, LPS can also induce endothelial cell apoptosis through the MAPK signaling pathway [73]. Therefore, in response to LPS-induced peripheral inflammation, the integrity of brain endothelial cells is impaired, and the BBB function is damaged, further exacerbating the entry of inflammatory factors into the brain to induce a central inflammatory response.

## 4. Stress and Amygdala

Stress-induced inflammatory processes and microglia-activation-related neuroinflammation have numerous effects on the structure and function of neurons in the brain and are associated with most pathophysiological diseases in the CNS [23,45]. Increasing evidence has consistently proved that the amygdala is a kernel brain region in mediating stress response. Over-activation of the amygdala is widely known as a fundamental process causing anxiety and depression [28,74]. In the resting state, amygdala activity is highly inhibited, which can avoid being activated by weak external stimuli and produce inappropriate emotional expression and abnormal behavior [75]. On the contrary, in pathological conditions (such as excessive stress exposure), the highly inhibitory tone of the amygdala is removed (disinhibition), leading to the hyperactivation of the amygdala, which in turn increases sensitivity to environmental stimulus signals even after long-term recovery [76,77]. The excessive activation of amygdala neurons caused by disinhibition is widely known as the neural basis of stress-induced neuropsychiatric disorders.

### 4.1. The Basic Anatomy of the Amygdala: Structure and Connectivity

The amygdala was first identified by Burdach in the early 19th century. Burdach originally described a group of cells that are now known as the basolateral complex [78]. Subsequently, many structures surrounding the basolateral complex were identified, now known as the amygdala complex. The amygdala complex contains more than 13 subregions, which can be distinguished according to their neuronal morphology, unique connectivity, physiology, and expression of specific molecular markers. It is usually divided into the basolateral amygdala (BLA), central amygdala (CeA), medial amygdala (MeA), and intercalated cell clusters [78]. Of note is that the BLA and CeA have attracted the most interest throughout the history of amygdala research, which are also the focus of this review. The BLA is constituted by the lateral amygdala (LA) and basal amygdala (BA), while the CeA is composed of the centrolateral (CeL) and centromedial (CeM) nuclei [79]. Developmentally, the BLA is a cortical-like structure consisting of 80% glutamatergic principal neurons and approximately 20% GABAergic inhibitory neurons [80,81]. In contrast, CeA is a striatum-like structure that primarily comprises GABAergic inhibitory neurons [82]. From a functional perspective, the BLA is widely known to encode the threat value of a stimulus, while the CeA is responsible for coordinating the behavioral output.

**Figure 3:**
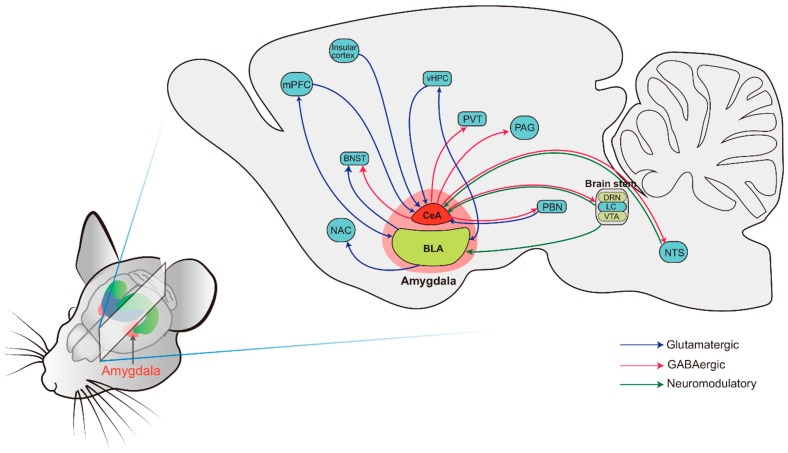
Schematic depicting illustrating the main input and output connections of the amygdala subnucleis. BLA: basolateral amygdala; BNST: bed nucleus of the stria terminalis; CeA: central amygdala; DRN: dorsal raphe nucleus; LC: locus coeruleus; mPFC: medial prefrontal cortex; NAc: nucleus accumbens; NTS: nucleus of the solitary tract; PAG: periaqueductal gray; PBN: parabrachial nucleus; PVT: paraventricular thalamus; vHPC: ventral hippocampus; VTA: ventral tegmental area.

The LA is traditionally known as the primary target of multimodal sensory information from the thalamus and cortex [83,84]. Upon receiving the information, the LA relays the integrated signal to the BA by its multi-axon lateral branches onto both glutamatergic projection neurons and GABAergic interneurons in the BA. This integrated information is then relayed to the CeA through dense efferent fibers. Notably, both the BLA and CeA receive dense projections and also have intensive projections to downstream brain regions, which are implicated in a host of behavioral and physiological responses, including anxiety, depression, fear, and other stress responses (Figure 3, refer to the review [76]).

The amygdala plays an essential role in regulating the activation of the HPA axis and SAM axis, which are hallmarks of the stress response [33]. It should be noted that unlike the inhibitory effect of the prefrontal cortex and hippocampus on HPA axis activation, the amygdala mainly acts as an activator of the HPA axis. In our recent review, we have described in detail the neural pathways of the amygdala subregions, including the BLA, CeA, and MeA, regulating the HPA axis [76], which will not be further described here. Of note is that the amygdala-triggered endocrine and autonomic responses to stress are not directly regulated by the HPA axis but are mediated by the middle diencephalon, such as through innervating BNST.

### 4.2. Effects of Stress on Amygdala Neuronal Function

Emerging evidence suggests that the hyperactivity of amygdala neurons is a fundamental cause of chronic stress-induced anxiety disorder and depression. Neuroimaging studies have shown that amygdala activation in patients with anxiety disorders is significantly higher than that in controls in response to the same stimulus [85], which is decreased after effective cognitive behavioral therapy [86]. In PTSD patients, functional magnetic resonance imaging (fMRI) showed exaggerated amygdala responses to facial expressions of fear, and the degree of amygdala activation is positively correlated to the severity of PTSD symptoms [87]. However, in patients with depression, both baseline amygdala activity and the amygdala response to emotional stimuli are increased [88,89], which is correlated with symptom severity [88]. Amygdala activity returned to normal after successful drug treatment [88]. In addition, adolescents with depression show increased amygdala activation [90].

The refined synaptic communication between excitatory and inhibitory synapses is essential for regulating neural activity and normal brain function [91]. An excitation/inhibition (E/I) imbalance in synaptic transmission and neural circuits leads to the increased excitability of amygdala neurons, which has been implicated in anxiety disorder, depression, and many other neuropsychiatric diseases [12,92]. Increased neuronal activity is mainly regulated by the following factors: The first one is the enhanced excitatory inputs onto recorded neurons, including the increased release of excitatory transmitters from presynaptic or the increased expression of postsynaptic excitatory receptors. It has been reported that chronic stress-induced changes in amygdala activity are associated with alterations in synaptic transmission onto amygdala neurons. For example, a large number of studies have reported that chronic stress enhances the excitatory synaptic transmission onto amygdala projection neurons by increasing the probability of excitatory neurotransmitter release at presynaptic terminals [93,94], which is consistent with the increased dendritic spine density in BLA neurons caused by chronic stress. The second one is the reduced presynaptic inhibitory transmitter release or decreased expression of the postsynaptic inhibitory receptor. A recent study has shown that chronic stress causes a reduction in excitatory, but not inhibitory, postsynaptic responses on PV-positive neurons in the BLA, which subsequently reduces GABAergic inhibitory input onto BLA projection neurons and results in an E/I imbalance in the amygdala [95]. However, our recent studies have found that chronic stress has no significant effect on inhibitory synaptic transmission onto BLA projection neurons [93,96]. The exact mechanisms underlying these inconsistent findings are unclear; it may be due to the different stress models and developmental stages of animals used. In addition to reducing the inhibitory effect mediated by the synaptic transmission form of BLA PNs, chronic stress also markedly weakens the inhibitory effect mediated by non-synaptic transmission, which significantly reduces the tonic inhibitory current recorded on BLA PNs, and then weakens the GABAergic suppression of the neuronal activity of BLA PNs [75]. The third one is the increased intrinsic neuronal excitability of neurons. Chronic stress significantly increases the intrinsic excitability of BLA PNs, and this effect is mainly due to the decreased expression and function of calcium-activated small conductance potassium channels and hyperpolarization-activated cyclic nucleotide-gated ion channels [28]. Taken together, the increased excitatory synaptic transmission, decreased inhibitory synaptic transmission, and increased intrinsic neuronal excitability together contribute to the overactivation of the amygdala, which is then implicated in anxiety disorder and depression induced by chronic stress.

## 5. Amygdala Neuroinflammation and Psychiatric Disorders

Increasing evidence indicates that elevated pro-inflammatory cytokine levels caused by the recruitment of peripheral cytokines to the brain and microglia activation play an essential role in the development of stress-induced anxiety disorder and depression. The possible mechanisms may involve the regulation of neurotransmitter metabolism, the influence of neuroendocrine function, synaptic plasticity, and the neural circuitry of emotion [97,98]. For example, chronic unpredictable stress induces both anxiety-like and depressive-like behaviors while increasing the expression of amygdala inflammatory cytokines, including IL-1β, IL-6, and TNF-α [99] (Table 1). The increased cytokines can regulate the activity of amygdala neurons. After 21 consecutive days of chronic restraint stress, NLRP3 and IL-1β expressions in the BLA region of rats were significantly up-regulated, and c-Fos expression was also significantly increased [100]. Neuroimaging data collected from humans show that inflammation increases amygdala activity, and the increased amygdala stress response is related to the increase in inflammatory cytokines [101,102]. Participants who received low-dose endotoxin had increased IL-6 and TNF-α levels and led to greater amygdala activity in response to socially threatening images, which are associated with an enhanced feeling of social disconnection [101]. A double-blind randomized crossover study showed that volunteers who received a typhoid vaccination injection increased circulating IL-6 and induced mood deterioration; it also increased amygdala activation upon stimulation [102]. Studies have shown that restraint stress upregulates the expression of NLRP3, cleaved caspase-1, and IL-1β in the amygdala and the expression levels of these proteins are more significantly increased by stress in female mice [103], which is consistent with the higher incidence of anxiety disorder and depression in females than males. These results suggest that stress-related inflammation may be an important contributor to mental illness.

### 5.1. Amygdala Inflammation and Anxiety Disorders

Numerous studies have implicated that inflammation-related anxiety disorders are associated with the aberrant activation of amygdala neurons. For example, studies have shown that in an inflammatory pain mouse model induced by complete Freund’s adjuvant (CFA) injection, the anxiety-like behavior in mice was markedly increased, which is associated with the upregulation of the excitatory postsynaptic receptors NMDA receptor and AMPA receptor in the BLA, as well as the postsynaptic dense protein PSD95, while the expression of inhibitory receptors GABA_A_ α2 and GABA_A_ γ2 was significantly decreased [127]. However, opposite findings were reported, in that the levels of GABA_A_ α2 and GABA_A_ γ2 were dramatically increased in the BLA of CFA-treated mice [128]. In terms of synaptic transmission, CFA injection has been found to increase BLA excitatory synaptic transmission and decrease inhibitory synaptic transmission, as reflected by the increased frequency of spontaneous excitatory postsynaptic currents and the decreased frequency of spontaneous inhibitory postsynaptic currents [127]. While considering the neurotransmitters, CFA increased glutamate levels in the BLA and decreased GABA levels [122]. These findings suggest that the inflammation-induced disruption of the amygdala E/I balance is manifested by enhanced excitatory presynaptic release and upregulated excitatory postsynaptic receptors, together with the decreased inhibitory presynaptic release and reduced expression of inhibitory receptors, which may contribute to the hyperactivity of the amygdala and finally result in the occurrence of anxiety disorders [35]. However, in our recent study, we found that LPS challenge mainly increases the excitatory synaptic transmission on BLA projection neurons but has no effect on inhibitory synaptic transmission [12]. The AMPA/NMDA ratio (a measure of postsynaptic changes in synaptic strength) also remained unaltered [12]. The exact reasons for these inconsistent results are not fully understood. One possible explanation is that the difference may be due to various factors, such as different models, experimental procedures, and the processing time involved. It should be noted that plantar injection of CFA induces not only an inflammatory response but also chronic pain, which is widely used as an animal model of chronic inflammatory pain. In contrast, i.p. injection of LPS mainly induces an inflammatory response. Interestingly, similar to the LPS-induced effects on the BLA, chronic stress also increased the excitatory synaptic transmission onto BLA PNs but had no obvious effect on the inhibitory synaptic transmission or NMDA/AMPA current ratio [92,94]. Given the concept that chronic stress induces amygdala inflammation, it is likely that amygdala inflammation may play a critical role in the chronic stress-induced functional remodeling of BLA PNs and anxiety-like behavior.

The specific mechanisms of how inflammation affects the BLA neuronal function and the occurrence of anxiety disorders are still vague. Such an effect may likely be related to the effect of cytokines on monoamine and glutamate. For example, studies showed that neonatal LPS treatment reduced TGF-β1 expression in the BLA during adulthood, which lead to the down-regulation of GABA_A_Rα2 subunit expression and GABA-induced current density, and ultimately caused the disruption of the E/I balance and the shift toward excitation, resulting in anxiety disorder [111]. Therefore, it is expected that anxiety-like behavior and the hyperactivity of the HPA axis induced by inflammation may be due to the increased neuron excitability caused by the decrease in the GABAergic inhibitory current in the BLA. Another study found that in LPS-induced anxiety models, IL33 expression was significantly upregulated in the BLA, while overexpression of IL33 in astrocytes inhibited BDNF expression through the NF-κB signaling pathway, affecting the synaptic transmission of GABA and causing anxiety disorders [112]. In addition, feeding on a chronic high-fat diet can promote anxiety-like behavior by increasing the expression of amygdala dopamine and pro-inflammatory cytokines TNF-α and IL-1β [124], while the local infusion of TNF-α-neutralizing antibody infliximab in the BLA reversed anxiety-like behavior in mice with persistent inflammatory pain [121]. In vitro patch-clamp recordings showed that TNF-α significantly enhanced AMPA receptor-mediated glutamate excitatory synaptic transmission and inhibited GABAA receptor-mediated BLA inhibitory synaptic transmission [121]. Furthermore, chemokine CXCL12, which has been considered a standard pro-inflammatory molecule for a long time, may also contribute to inflammatory-related anxiety as supported by the evidence that an LPS challenge induced chemokine CXCL12 production in the amygdala through astrocyte activation, while microinjection of CXCL12 into the amygdala is sufficient to induce anxious-like behavior in mice. In addition, CXCL12 enhances glutamatergic transmission by increasing the frequency of sEPSC in the BLA [129]. These findings provide direct evidence showing that pro-inflammatory molecules in the BLA contribute to the development of anxiety by systemic inflammatory stress, which also may be a potential therapeutic target for inflammatory anxiety.

The CeA also plays a vital role in regulating the immune stress response. The CeA was activated in male and female rats after LPS injection 14 days after birth [130]. CeA lesion significantly reduced the systemic injection of IL-1β-induced ACTH secretion, CRH in the hypothalamus, and c-Fos gene expression in oxytocin cells [131]. Similarly, bilateral CeA damage also attenuated the HPA axis response caused by HSV-1 infection, as well as fever, hyperactivity, and aggression [132]. It should be noted that different stress modes have distinct effects on CeA activation. For example, homeostasis disruption and systemic stressors (such as inflammatory stimulation and bleeding) activate the CeA [133,134], while psychological stressors such as restrain stress do not activate the CeA [131,135,136]. In contrast, the MeA is preferentially activated by psychological stressors. MeA damage reduced the IL1β response to restraint stress but not to homeostasis and systemic stressors [135].

### 5.2. Amygdala Inflammation and Depression

Although the vast majority of studies revealing the role of the amygdala in modulating emotional function have been focused on fear and anxiety, recent studies have shown that amygdala inflammation has an undoubtful function in depression. For example, intravenous or subcutaneous IL-1β injection increased depression-like behavior in mice and enhanced the expression of pro-inflammatory cytokines TNF-α and IL-6 in the amygdala, which is regulated by the CORT/GR system. Furthermore, serum corticosterone levels were markedly increased with IL-1β injection, while GR inhibitor RU486 reduced IL-1β-induced depression-like behavior and TNF-α and IL-6 expression [120]. In addition, chronic social stress (a combination of mild prenatal stress, mild maternal separation stress, and mild social frustration stress) induced depressive-like behavior, which is accompanied by the activation of amygdala microglia and increased expression of inflammatory factors IL-17, TNF-α, IL-1β, IL18, and IL-6. Interestingly, peripheral intraperitoneal injection of IL-17 antibody to block IL-17 activity inhibited stress-induced BLA microglia activation and alleviated depression-like behavior [105]. In addition, LPS-induced inflammation increases CeA activity in animals [137], which is associated with an increase in depressive-like behavior [137] and a reduction in certain social behaviors, such as grooming, sniffing, and close following with an interaction partner [138]. Blocking the activity in the amygdala by using a reversible lesion eliminates this inflammation-induced social withdrawal behavior [138].

In mammals, the downregulation of serotonin (5-HT) levels in the CNS has been implicated in the pathophysiology of depression. With the development of the cytokine theory of depression, it has been well documented that pro-inflammatory cytokines, including IL-1β, IFN, and TNF can reduce the bioavailability of neurotransmitters such as serotonin (5-HT) [139]. The synthetic precursor of 5-HT, tryptophan, is metabolized by the kynurenine pathway (KP) and 5-HT pathway [140]. Indeed, over 90% of tryptophan has been metabolized via the KP in mammals [141]. In the KP, indoleamine-2,3-dioxygenase (IDO) is a rate-limiting enzyme that catalyzes the decomposition and metabolism of tryptophan [140]. It should be noted that IDO is an inflammatory-inducible enzyme, which can be induced by a variety of inflammatory factors, such as IFNγ and TNFα [142,143]. Activation of the body’s immune system causes excessive secretion of cytokines, induces the activation of IDO, and increases the metabolism of tryptophan along the KP pathway, thereby competitively antagonizing the biosynthesis process of 5-HT and reducing the central 5-HT neurotransmission. Therefore, IDO plays an essential role in the cytokine theory of depression.

A large amount of evidence has shown that inflammatory response is accompanied by IDO dysfunction in depression [143,144]. Early-life stress from allergic dermatitis increases susceptibility to depression induced by systemic inflammation, which is accompanied by increased activation of microglia and cytokine expression in the amygdala, as well as upregulated expressions of IDO [118]. High IDO levels indicate that 5-HT metabolism may be accelerated, thus reducing the 5-HT level. The exact mechanism by which inflammation induces upregulation of IDO expression in the amygdala remains unclear. However, it should be noted that pro-inflammatory cytokines, such as IL-1β, can cross the BBB and trigger neuroinflammation by activating microglia. It has been reported that activated microglia release pro-inflammatory cytokines that reduce the synthesis of 5-HT, dopamine, and norepinephrine in the limbic system by activating IDO and mitogen-activated protein kinase (MAPK) in the inflammatory signaling pathway [98,145,146]. Interestingly, numerous studies have shown that inflammatory responses increase amygdala IL-1β expression and activate MAPK pathways (increased phosphorylation of JNK, ERK1/2, and P38).

### 5.3. Inflammation and the Amygdala Neural Circuit

The structural and functional connectivity between the amygdala and other emotion-related brain regions, such as the mPFC, hippocampus, and anterior cingulate cortex, play crucial roles in stress-induced anxiety and depressive behaviors [147,148,149]. Among them, the enhanced dmPFC-amygdala circuit has been widely shown to be implicated in inflammatory-related psychiatric disorders. For example, increased amygdala activity is associated with inflammation when exposed to social stress. Individuals who exhibit stronger coupling between the amygdala and dmPFC display higher inflammatory responses when faced with stressors [150]. Our recent study demonstrated that LPS causes anxiety and depression-like behaviors accompanied by an enhanced activity of the dmPFC-BLA circuits [12]. In addition, allergic inflammation leads to enhanced functional connectivity within the mPFC-amygdala circuit, while disrupting the dynamic interactions of the mPFC-amygdala circuit may promote anxiety-related behaviors with asthma [151]. Furthermore, a recent study from the same lab showed that allergic inflammation induced an increase in neuronal activity, and functional connectivity of the ACC-BLA circuit was correlated with the level of anxiety [152]. Inflammation-associated mood change reduced the connectivity of sACC to the amygdala, which was modulated by peripheral IL-6 [102].

It is worth noting that many studies have shown that the abnormal activation of the mPFC-BLA-vHPC circuit contributes to chronic stress-induced anxiety [77,92,96,153], and depressive-like behavior [154]. Specifically, chronic stress increases the glutamate release from dmPFC presynaptic terminals in the BLA, which enhances the excitatory synaptic transmission efficiency of BLA neurons [92]. Interestingly, chronic stress-induced abnormal increase in BLA neuron activity only occurred in BLA neurons projected to the vHPC [96,153]. The specific mechanism of stress-induced changes in the amygdala circuit function remains unclear. Considering the effects of inflammatory cytokines on BLA neuronal function and connectivity, it is expected that chronic stress-induced functional remodeling of the BLA circuit may be due to the neuroinflammation caused by stress.

There is also evidence showing that changes within CeA circuit are implicated in inflammatory-related mental disease. For example, a recent study showed that in a mouse model of sepsis induced by intraperitoneal infection, the mice exhibited anxiety-like behavior and exaggerated fear memories, which are similar to anxiety and PTSD-like symptoms seen in human sepsis survivors [155]. It was found that sepsis induces the acute pathological activation of CeA-specific neuron types that project to the ventral BNST and transient and targeted silencing of this subpopulation using chemogenetic methods during the acute phase of sepsis can prevent the subsequent development of anxiety-related behaviors [155]. Considering inflammatory chronic pain increases the risk of depression, Zhou and colleagues identified a novel pathway involving 5-HT projections from the dorsal raphe nucleus to somatostatin-expressing interneurons in the CeA, and then to the lateral habenula for comorbid depressive symptoms in chronic pain [156]. In addition to the evidence that microglia-mediated synaptic engulfment in the CeA contributes to visceral pain [157], it is suggested that the CeA may act as a probable convergent point of chronic pain and depression. Interestingly, in addition to the well-established neuroendocrine-mediated pathway, by which stress regulates immune responses via the systemic release of neuroendocrine systemic mediators (CRH/ACTH/corticosterone), a recent study identified a direct descending neuronal circuit from the CeA and paraventricular CRH neurons into the spleen, which is activated in response to stress. Pharmacogenetic activation of this pathway increases plasma cell abundance after immunization [158]. This finding suggested a novel role of the amygdala in regulating the immune system.

## 6. Conclusions and Prospects

A growing body of evidence shows that stress-mediated immune response and neuroinflammation contribute to anxiety disorder and depression upon stress exposure, which is closely related to the structural and functional remodeling of the amygdala. Nevertheless, there are still a series of unsolved questions that need further investigation. Foremost, a critical question that needs to be addressed is how amygdala inflammation is incorporated into the development of depression and anxiety disorders. Although numerous studies have indicated that inflammation-induced anxiety and depression are accompanied by microglia activation and inflammatory gene expression in the amygdala, the causal relationship between amygdala inflammation and the development of psychiatric disorders is still vague. Second, there is still conflicting evidence of stress in the inflammatory response and neuronal function in the amygdala. For example, footshock stress has been shown to change the activity of BLA neurons in the amygdala to mediate the occurrence of anxiety disorder, while a recent study found that footshock did not induce inflammation in the amygdala, thus raising an intriguing question that the inflammation-induced functional remodeling and behavioral changes in the amygdala caused by psychogenic stress are associated with the stress type. More comprehensive studies are needed in the future to help better understand the immune-neuro-psychological role of the amygdala. Third, since it has been widely documented that communication between neurons and microglia is highly coordinated, the possible interaction between these cells in the amygdala and the potential mechanism in the pathology of depression and anxiety disorder is a venue meriting further exploration. Fourth, the distinct neural circuits in the amygdala are implicated in different emotional behavior outputs; whether those circuits are differentially affected by inflammation remains an open question, which adds another layer of complexity toward understanding the exact role that amygdala inflammation plays in stress-induced depression and anxiety disorder. Further exploration of these questions will improve our understanding of the pathogenesis of mental diseases and help to develop better treatments to improve the quality of life of individuals suffering from anxiety disorders and depression.

## Figures and Tables

**Table 1:** Effects of stress on inflammatory cytokines in the amygdala.

Stress Type	Inflammatory in Amygdala	Species	Reference
Chronic unpredictable mild stress	IL-1β↑ ^1^, IL-6↑, TNF-α↑	Mouse	[99]
Chronic unpredictable mild stress	IL-1β↑, IL-10↑	Hamsters	[104]
Chronic restrain stress	BLA: NLRP3↑, IL-1β↑	Rat	[100]
Cumulative mild stress	TGF-β↑, IL-1β↑, IL-17↑, IL-18↑, IL-6↑	Mouse	[105]
Chronic social defeat stress	BLA: IL-1β↑, IL-6↑, TNF-α↑, IL-12↑	Mouse	[106]
Modified social defeat stress	IL-6 – ^2^	Rat	[107]
Foot-shock stress	IL-4 –, IL-10 –, IL-1β –, TNF-α –, INF-γ –, IL-6↓ ^3^	Mouse	[108]
Forced swim stress	TNF-α↑, IL-6–, IL-1β –	Rats	[109]
Physical restraint stress with brief underwater submersion, and predator odor stress	IL-6↑, IL-1β↑, Caspase 1↑, NLRP3↑	Mouse	[103]
Chronic prostatitis/chronic pelvic pain syndrome	BLA: IL-1R↓, IL-4R↓, IL-13R↓, TNFR↓	Rats	[110]
LPS	BLA: TGF-β1↓, TNF-α↑, IL-1β↑; IL-33↑	Mouse	[111,112]
LPS	IL-6↑, IL-1β↑, TNF-α↑, IL-10↑; IL-6↑, IL-1β↑, vWF↑; IL-6↑, IL-1β↑, TNF-α↑; IL-6↑, IL-1β↑	Mouse	[113,114,115,116,117]
LPS + oxazolone (Ox)-induced AD model	IL-6↑	Mouse	[118]
Central LPS infusion	IL-1β↑, TNF-α↑	Rats	[119]
Intracerebroventricular(i.c.v.) injection of IL-1β	IL-6↑, TNF-α↑	Mouse	[120]
Injection of complete Freund’s adjuvant (CFA)	BLA: TNF-α↑IL-1β↑, IL-6↑, TNF-α↑	Mouse	[121,122]
High-fat diet	IL-1β↑, TNF-α↑; TNF-α↑	Mouse	[123,124]
High-fat diet	IL-6↑, CD11b –; IL-1Ra↑; IL-6 –, CD11b –, IL-1Rα↓	Rats	[125,126]

^1^ ↑ indicates up-regulated expression level; ^2^ – indicates no change; ^3^ ↓ indicates down-regulated expression level.

## Data Availability

Not applicable.

## References

[1] Disease G.B.D., Injury I., Prevalence C. (2017). Global, regional, and national incidence, prevalence, and years lived with disability for 328 diseases and injuries for 195 countries, 1990–2016: A systematic analysis for the Global Burden of Disease Study 2016. Lancet.

[2] Almeida-Filho N., Lessa I., Magalhaes L., Arauho M.J., Aquino E., de Jesus M.J. (2007). Co-occurrence patterns of anxiety, depression and alcohol use disorders. Eur. Arch. Psychiatry Clin. Neurosci..

[3] de Kloet E.R., Joels M., Holsboer F. (2005). Stress and the brain: From adaptation to disease. Nat. Rev. Neurosci..

[4] Collaborators C.-M.D. (2021). Global prevalence and burden of depressive and anxiety disorders in 204 countries and territories in 2020 due to the COVID-19 pandemic. Lancet.

[5] Delgado P.L. (2000). Depression: The case for a monoamine deficiency. J. Clin. Psychiatry.

[6] Liu B., Liu J., Wang M., Zhang Y., Li L. (2017). From serotonin to neuroplasticity: Evolvement of theories for major depressive disorder. Front. Cell. Neurosci..

[7] Jakobsen J.C., Katakam K.K., Schou A., Hellmuth S.G., Stallknecht S.E., Leth-Moller K., Iversen M., Banke M.B., Petersen I.J., Klingenberg S.L. (2017). Selective serotonin reuptake inhibitors versus placebo in patients with major depressive disorder. A systematic review with meta-analysis and Trial Sequential Analysis. BMC Psychiatry.

[8] Westfall S., Caracci F., Estill M., Frolinger T., Shen L., Pasinetti G.M. (2021). Chronic stress-induced depression and anxiety priming modulated by gut-brain-axis immunity. Front. Immunol..

[9] Risbrough V.B., Vaughn M.N., Friend S.F. (2022). Role of inflammation in traumatic brain injury-associated risk for neuropsychiatric disorders: State of the evidence and where do we go from here. Biol. Psychiatry.

[10] Milaneschi Y., Kappelmann N., Ye Z., Lamers F., Moser S., Jones P.B., Burgess S., Penninx B., Khandaker G.M. (2021). Association of inflammation with depression and anxiety: Evidence for symptom-specificity and potential causality from UK Biobank and NESDA cohorts. Mol. Psychiatry.

[11] Slavich G.M., Irwin M.R. (2014). From stress to inflammation and major depressive disorder: A social signal transduction theory of depression. Psychol. Bull..

[12] Zheng Z.H., Tu J.L., Li X.H., Hua Q., Liu W.Z., Liu Y., Pan B.X., Hu P., Zhang W.H. (2021). Neuroinflammation induces anxiety- and depressive-like behavior by modulating neuronal plasticity in the basolateral amygdala. Brain Behav. Immun..

[13] Kiecolt-Glaser J.K., Derry H.M., Fagundes C.P. (2015). Inflammation: Depression fans the flames and feasts on the heat. Am. J. Psychiatry.

[14] Beurel E., Toups M., Nemeroff C.B. (2020). The bidirectional relationship of depression and inflammation: Double trouble. Neuron.

[15] Euesden J., Danese A., Lewis C.M., Maughan B. (2017). A bidirectional relationship between depression and the autoimmune disorders—New perspectives from the National Child Development Study. PLoS ONE.

[16] Dickens C., McGowan L., Clark-Carter D., Creed F. (2002). Depression in rheumatoid arthritis: A systematic review of the literature with meta-analysis. Psychosom. Med..

[17] Patten S.B., Marrie R.A., Carta M.G. (2017). Depression in multiple sclerosis. Int. Rev. Psychiatry.

[18] Robinson R.G., Jorge R.E. (2016). Post-stroke depression: A review. Am. J. Psychiatry.

[19] Machado M.O., Oriolo G., Bortolato B., Kohler C.A., Maes M., Solmi M., Grande I., Martin-Santos R., Vieta E., Carvalho A.F. (2017). Biological mechanisms of depression following treatment with interferon for chronic hepatitis C: A critical systematic review. J. Affect. Disord..

[20] Chiu W.C., Su Y.P., Su K.P., Chen P.C. (2017). Recurrence of depressive disorders after interferon-induced depression. Transl. Psychiatry.

[21] Griffiths C.E.M., Fava M., Miller A.H., Russell J., Ball S.G., Xu W., Acharya N., Rapaport M.H. (2017). Impact of ixekizumab treatment on depressive symptoms and systemic inflammation in patients with moderate-to-severe psoriasis: An integrated analysis of three phase 3 clinical studies. Psychother. Psychosom..

[22] Felger J.C. (2018). Imaging the role of inflammation in mood and anxiety-related disorders. Curr. Neuropharmacol..

[23] Leonard B.E. (2018). Inflammation and depression: A causal or coincidental link to the pathophysiology?. Acta Neuropsychiatr..

[24] Russo S.J., Nestler E.J. (2013). The brain reward circuitry in mood disorders. Nat. Rev. Neurosci..

[25] Price J.L., Drevets W.C. (2010). Neurocircuitry of mood disorders. Neuropsychopharmacology.

[26] O’Neill P.K., Gore F., Salzman C.D. (2018). Basolateral amygdala circuitry in positive and negative valence. Curr. Opin. Neurobiol..

[27] Sah P. (2017). Fear, anxiety, and the amygdala. Neuron.

[28] Rosenkranz J.A., Venheim E.R., Padival M. (2010). Chronic stress causes amygdala hyperexcitability in rodents. Biol. Psychiatry.

[29] Zhang W., Rosenkranz J.A. (2012). Repeated restraint stress increases basolateral amygdala neuronal activity in an age-dependent manner. Neuroscience.

[30] Birbaumer N., Grodd W., Diedrich O., Klose U., Erb M., Lotze M., Schneider F., Weiss U., Flor H. (1998). fMRI reveals amygdala activation to human faces in social phobics. Neuroreport.

[31] Phan K.L., Fitzgerald D.A., Nathan P.J., Tancer M.E. (2006). Association between amygdala hyperactivity to harsh faces and severity of social anxiety in generalized social phobia. Biol. Psychiatry.

[32] O’Connor D.B., Thayer J.F., Vedhara K. (2021). Stress and health: A review of psychobiological processes. Annu. Rev. Psychol..

[33] Ulrich-Lai Y.M., Herman J.P. (2009). Neural regulation of endocrine and autonomic stress responses. Nat. Rev. Neurosci..

[34] McEwen B.S., Gianaros P.J. (2010). Central role of the brain in stress and adaptation: Links to socioeconomic status, health, and disease. Ann. N. Y. Acad. Sci..

[35] McEwen B.S. (1998). Protective and damaging effects of stress mediators. N. Engl. J. Med..

[36] Cao P., Chen C., Liu A., Shan Q., Zhu X., Jia C., Peng X., Zhang M., Farzinpour Z., Zhou W. (2021). Early-life inflammation promotes depressive symptoms in adolescence via microglial engulfment of dendritic spines. Neuron.

[37] Miller G.E., Cohen S., Ritchey A.K. (2002). Chronic psychological stress and the regulation of pro-inflammatory cytokines: A glucocorticoid-resistance model. Health Psychol..

[38] Cohen S., Janicki-Deverts D., Miller G.E. (2007). Psychological stress and disease. JAMA.

[39] Gordan R., Gwathmey J.K., Xie L.H. (2015). Autonomic and endocrine control of cardiovascular function. World J. Cardiol..

[40] Hasko G., Szabo C. (1998). Regulation of cytokine and chemokine production by transmitters and co-transmitters of the autonomic nervous system. Biochem. Pharmacol..

[41] Pavlov V.A., Tracey K.J. (2012). The vagus nerve and the inflammatory reflex—Linking immunity and metabolism. Nat. Rev. Endocrinol..

[42] Pavlov V.A., Wang H., Czura C.J., Friedman S.G., Tracey K.J. (2003). The cholinergic anti-inflammatory pathway: A missing link in neuroimmunomodulation. Mol. Med..

[43] Borovikova L.V., Ivanova S., Nardi D., Zhang M., Yang H., Ombrellino M., Tracey K.J. (2000). Role of vagus nerve signaling in CNI-1493-mediated suppression of acute inflammation. Auton Neurosci..

[44] Frost J.L., Schafer D.P. (2016). Microglia: Architects of the developing nervous system. Trends Cell Biol..

[45] Li Q., Barres B.A. (2018). Microglia and macrophages in brain homeostasis and disease. Nat. Rev. Immunol..

[46] Stratoulias V., Venero J.L., Tremblay M.E., Joseph B. (2019). Microglial subtypes: Diversity within the microglial community. EMBO J..

[47] Dheen S.T., Kaur C., Ling E.A. (2007). Microglial activation and its implications in the brain diseases. Curr. Med. Chem..

[48] Stellwagen D., Malenka R.C. (2006). Synaptic scaling mediated by glial TNF-alpha. Nature.

[49] Yirmiya R., Goshen I. (2011). Immune modulation of learning, memory, neural plasticity and neurogenesis. Brain Behav. Immun..

[50] Stephan A.H., Barres B.A., Stevens B. (2012). The complement system: An unexpected role in synaptic pruning during development and disease. Annu. Rev. Neurosci..

[51] Dantzer R., O’Connor J.C., Freund G.G., Johnson R.W., Kelley K.W. (2008). From inflammation to sickness and depression: When the immune system subjugates the brain. Nat. Rev. Neurosci..

[52] Frank M.G., Baratta M.V., Sprunger D.B., Watkins L.R., Maier S.F. (2007). Microglia serve as a neuroimmune substrate for stress-induced potentiation of CNS pro-inflammatory cytokine responses. Brain Behav. Immun..

[53] Hinwood M., Morandini J., Day T.A., Walker F.R. (2012). Evidence that microglia mediate the neurobiological effects of chronic psychological stress on the medial prefrontal cortex. Cereb. Cortex.

[54] Kreisel T., Frank M.G., Licht T., Reshef R., Ben-Menachem-Zidon O., Baratta M.V., Maier S.F., Yirmiya R. (2014). Dynamic microglial alterations underlie stress-induced depressive-like behavior and suppressed neurogenesis. Mol. Psychiatry.

[55] Steiner J., Walter M., Gos T., Guillemin G.J., Bernstein H.G., Sarnyai Z., Mawrin C., Brisch R., Bielau H., Meyer zu Schwabedissen L. (2011). Severe depression is associated with increased microglial quinolinic acid in subregions of the anterior cingulate gyrus: Evidence for an immune-modulated glutamatergic neurotransmission?. J. Neuroinflammation.

[56] Schnieder T.P., Trencevska I., Rosoklija G., Stankov A., Mann J.J., Smiley J., Dwork A.J. (2014). Microglia of prefrontal white matter in suicide. J. Neuropathol. Exp. Neurol..

[57] Calcia M.A., Bonsall D.R., Bloomfield P.S., Selvaraj S., Barichello T., Howes O.D. (2016). Stress and neuroinflammation: A systematic review of the effects of stress on microglia and the implications for mental illness. Psychopharmacology.

[58] Johnson J.D., Cortez V., Kennedy S.L., Foley T.E., Hanson H., Fleshner M. (2008). Role of central beta-adrenergic receptors in regulating proinflammatory cytokine responses to a peripheral bacterial challenge. Brain Behav. Immun..

[59] Johnson J.D., Zimomra Z.R., Stewart L.T. (2013). Beta-adrenergic receptor activation primes microglia cytokine production. J. Neuroimmunol..

[60] McNamee E.N., Griffin E.W., Ryan K.M., Ryan K.J., Heffernan S., Harkin A., Connor T.J. (2010). Noradrenaline acting at beta-adrenoceptors induces expression of IL-1beta and its negative regulators IL-1ra and IL-1RII, and drives an overall anti-inflammatory phenotype in rat cortex. Neuropharmacology.

[61] Hoogland I.C., Houbolt C., van Westerloo D.J., van Gool W.A., van de Beek D. (2015). Systemic inflammation and microglial activation: Systematic review of animal experiments. J. Neuroinflammation.

[62] Konsman J.P., Parnet P., Dantzer R. (2002). Cytokine-induced sickness behaviour: Mechanisms and implications. Trends Neurosci..

[63] Cao C., Matsumura K., Yamagata K., Watanabe Y. (1996). Endothelial cells of the rat brain vasculature express cyclooxygenase-2 mRNA in response to systemic interleukin-1 beta: A possible site of prostaglandin synthesis responsible for fever. Brain Res..

[64] Bonaz B., Sinniger V., Pellissier S. (2017). The vagus nerve in the neuro-immune axis: Implications in the pathology of the gastrointestinal tract. Front. Immunol..

[65] Ek M., Kurosawa M., Lundeberg T., Ericsson A. (1998). Activation of vagal afferents after intravenous injection of interleukin-1beta: Role of endogenous prostaglandins. J. Neurosci..

[66] Dantzer R., Kelley K.W. (2007). Twenty years of research on cytokine-induced sickness behavior. Brain Behav. Immun..

[67] Wan W., Janz L., Vriend C.Y., Sorensen C.M., Greenberg A.H., Nance D.M. (1993). Differential induction of c-Fos immunoreactivity in hypothalamus and brain stem nuclei following central and peripheral administration of endotoxin. Brain Res. Bull..

[68] Galea I. (2021). The blood-brain barrier in systemic infection and inflammation. Cell. Mol. Immunol..

[69] Kadry H., Noorani B., Cucullo L. (2020). A blood-brain barrier overview on structure, function, impairment, and biomarkers of integrity. Fluids Barriers CNS.

[70] Wang X., Xue G.X., Liu W.C., Shu H., Wang M., Sun Y., Liu X., Sun Y.E., Liu C.F., Liu J. (2017). Melatonin alleviates lipopolysaccharide-compromised integrity of blood-brain barrier through activating AMP-activated protein kinase in old mice. Aging Cell.

[71] Qin L.H., Huang W., Mo X.A., Chen Y.L., Wu X.H. (2015). LPS induces occludin dysregulation in cerebral microvascular endothelial cells via MAPK signaling and augmenting MMP-2 levels. Oxid. Med. Cell. Longev..

[72] Salkeni M.A., Lynch J.L., Otamis-Price T., Banks W.A. (2009). Lipopolysaccharide impairs blood-brain barrier P-glycoprotein function in mice through prostaglandin- and nitric oxide-independent pathways. J. Neuroimmune Pharmacol..

[73] Karahashi H., Michelsen K.S., Arditi M. (2009). Lipopolysaccharide-induced apoptosis in transformed bovine brain endothelial cells and human dermal microvessel endothelial cells: The role of JNK. J. Immunol..

[74] van den Bulk B.G., Meens P.H., van Lang N.D., de Voogd E.L., van der Wee N.J., Rombouts S.A., Crone E.A., Vermeiren R.R. (2014). Amygdala activation during emotional face processing in adolescents with affective disorders: The role of underlying depression and anxiety symptoms. Front. Hum. Neurosci..

[75] Liu Z.P., Song C., Wang M., He Y., Xu X.B., Pan H.Q., Chen W.B., Peng W.J., Pan B.X. (2014). Chronic stress impairs GABAergic control of amygdala through suppressing the tonic GABAA receptor currents. Mol. Brain.

[76] Zhang W.H., Zhang J.Y., Holmes A., Pan B.X. (2021). Amygdala circuit substrates for stress adaptation and adversity. Biol. Psychiatry.

[77] Qin X., Pan H.Q., Huang S.H., Zou J.X., Zheng Z.H., Liu X.X., You W.J., Liu Z.P., Cao J.L., Zhang W.H. (2022). GABAA(δ) receptor hypofunction in the amygdala-hippocampal circuit underlies stress-induced anxiety. Sci. Bull..

[78] Sah P., Faber E.S., Lopez De Armentia M., Power J. (2003). The amygdaloid complex: Anatomy and physiology. Physiol. Rev..

[79] Gilpin N.W., Herman M.A., Roberto M. (2015). The central amygdala as an integrative hub for anxiety and alcohol use disorders. Biol. Psychiatry.

[80] Carlsen J., Heimer L. (1988). The basolateral amygdaloid complex as a cortical-like structure. Brain Res..

[81] Keifer O.P., Hurt R.C., Ressler K.J., Marvar P.J. (2015). The physiology of fear: Reconceptualizing the role of the central amygdala in fear learning. Physiology.

[82] McDonald A.J. (1982). Cytoarchitecture of the central amygdaloid nucleus of the rat. J. Comp. Neurol..

[83] LeDoux J.E., Cicchetti P., Xagoraris A., Romanski L.M. (1990). The lateral amygdaloid nucleus: Sensory interface of the amygdala in fear conditioning. J. Neurosci..

[84] Tully K., Li Y., Tsvetkov E., Bolshakov V.Y. (2007). Norepinephrine enables the induction of associative long-term potentiation at thalamo-amygdala synapses. Proc. Natl. Acad. Sci. USA.

[85] Thomas K.M., Drevets W.C., Dahl R.E., Ryan N.D., Birmaher B., Eccard C.H., Axelson D., Whalen P.J., Casey B.J. (2001). Amygdala response to fearful faces in anxious and depressed children. Arch. Gen. Psychiatry.

[86] Felmingham K., Kemp A., Williams L., Das P., Hughes G., Peduto A., Bryant R. (2007). Changes in anterior cingulate and amygdala after cognitive behavior therapy of posttraumatic stress disorder. Psychol. Sci..

[87] Shin L.M., Wright C.I., Cannistraro P.A., Wedig M.M., McMullin K., Martis B., Macklin M.L., Lasko N.B., Cavanagh S.R., Krangel T.S. (2005). A functional magnetic resonance imaging study of amygdala and medial prefrontal cortex responses to overtly presented fearful faces in posttraumatic stress disorder. Arch. Gen. Psychiatry.

[88] Drevets W.C., Bogers W., Raichle M.E. (2002). Functional anatomical correlates of antidepressant drug treatment assessed using PET measures of regional glucose metabolism. Eur. Neuropsychopharmacol..

[89] Hamilton J.P., Gotlib I.H. (2008). Neural substrates of increased memory sensitivity for negative stimuli in major depression. Biol. Psychiatry.

[90] Yang T.T., Simmons A.N., Matthews S.C., Tapert S.F., Frank G.K., Max J.E., Bischoff-Grethe A., Lansing A.E., Brown G., Strigo I.A. (2010). Adolescents with major depression demonstrate increased amygdala activation. J. Am. Acad. Child Adolesc. Psychiatry.

[91] Froemke R.C. (2015). Plasticity of cortical excitatory-inhibitory balance. Annu. Rev. Neurosci..

[92] Liu W.Z., Zhang W.H., Zheng Z.H., Zou J.X., Liu X.X., Huang S.H., You W.J., He Y., Zhang J.Y., Wang X.D. (2020). Identification of a prefrontal cortex-to-amygdala pathway for chronic stress-induced anxiety. Nat. Commun..

[93] Qin X., Liu X.X., Wang Y., Wang D., Song Y., Zou J.X., Pan H.Q., Zhai X.Z., Zhang Y.M., Zhang Y.B. (2021). Early life stress induces anxiety-like behavior during adulthood through dysregulation of neuronal plasticity in the basolateral amygdala. Life Sci..

[94] Qin X., He Y., Wang N., Zou J.X., Zhang Y.M., Cao J.L., Pan B.X., Zhang W.H. (2019). Moderate maternal separation mitigates the altered synaptic transmission and neuronal activation in amygdala by chronic stress in adult mice. Mol. Brain.

[95] Luo Z.Y., Huang L., Lin S., Yin Y.N., Jie W., Hu N.Y., Hu Y.Y., Guan Y.F., Liu J.H., You Q.L. (2020). Erbin in amygdala parvalbumin-positive neurons modulates anxiety-like behaviors. Biol. Psychiatry.

[96] Zhang J.Y., Liu T.H., He Y., Pan H.Q., Zhang W.H., Yin X.P., Tian X.L., Li B.M., Wang X.D., Holmes A. (2019). Chronic stress remodels synapses in an amygdala circuit-specific manner. Biol. Psychiatry.

[97] Miller A.H., Haroon E., Raison C.L., Felger J.C. (2013). Cytokine targets in the brain: Impact on neurotransmitters and neurocircuits. Depress. Anxiety.

[98] Miller A.H., Maletic V., Raison C.L. (2009). Inflammation and its discontents: The role of cytokines in the pathophysiology of major depression. Biol. Psychiatry.

[99] Nazir S., Farooq R.K., Nasir S., Hanif R., Javed A. (2022). Therapeutic effect of Thymoquinone on behavioural response to UCMS and neuroinflammation in hippocampus and amygdala in BALB/c mice model. Psychopharmacology.

[100] Meng Y., Zhuang L., Xue Q., Zhang J., Yu B. (2021). NLRP3-mediated Neuroinflammation Exacerbates Incisional Hyperalgesia and Prolongs Recovery After Surgery in Chronic Stressed Rats. Pain Physician.

[101] Inagaki T.K., Muscatell K.A., Irwin M.R., Cole S.W., Eisenberger N.I. (2012). Inflammation selectively enhances amygdala activity to socially threatening images. Neuroimage.

[102] Harrison N.A., Brydon L., Walker C., Gray M.A., Steptoe A., Critchley H.D. (2009). Inflammation causes mood changes through alterations in subgenual cingulate activity and mesolimbic connectivity. Biol. Psychiatry.

[103] Ghosh S., Mohammed Z., Singh I. (2021). Bruton’s tyrosine kinase drives neuroinflammation and anxiogenic behavior in mouse models of stress. J. Neuroinflammation.

[104] Avolio E., Fazzari G., Mele M., Alo R., Zizza M., Jiao W., Di Vito A., Barni T., Mandala M., Canonaco M. (2017). Unpredictable chronic mild stress paradigm established effects of pro- and anti-inflammatory cytokine on neurodegeneration-linked depressive states in hamsters with brain endothelial damages. Mol. Neurobiol..

[105] Kim J., Suh Y.H., Chang K.A. (2021). Interleukin-17 induced by cumulative mild stress promoted depression-like behaviors in young adult mice. Mol. Brain.

[106] Nozaki K., Ito H., Ohgidani M., Yamawaki Y., Sahin E.H., Kitajima T., Katsumata S., Yamawaki S., Kato T.A., Aizawa H. (2020). Antidepressant effect of the translocator protein antagonist ONO-2952 on mouse behaviors under chronic social defeat stress. Neuropharmacology.

[107] Patki G., Solanki N., Atrooz F., Allam F., Salim S. (2013). Depression, anxiety-like behavior and memory impairment are associated with increased oxidative stress and inflammation in a rat model of social stress. Brain Res..

[108] Li S., Liao Y., Dong Y., Li X., Li J., Cheng Y., Cheng J., Yuan Z. (2021). Microglial deletion and inhibition alleviate behavior of post-traumatic stress disorder in mice. J. Neuroinflammation.

[109] Guan X.T., Lin W.J., Tang M.M. (2015). Comparison of stress-induced and LPS-induced depressive-like behaviors and the alterations of central proinflammatory cytokines mRNA in rats. Psych. J..

[110] Hu C., Yang H., Zhao Y., Chen X., Dong Y., Li L., Dong Y., Cui J., Zhu T., Zheng P. (2016). The role of inflammatory cytokines and ERK1/2 signaling in chronic prostatitis/chronic pelvic pain syndrome with related mental health disorders. Sci. Rep..

[111] Zhong H., Rong J., Yang Y., Liang M., Li Y., Zhou R. (2022). Neonatal inflammation via persistent TGF-beta1 downregulation decreases GABAAR expression in basolateral amygdala leading to the imbalance of the local excitation-inhibition circuits and anxiety-like phenotype in adult mice. Neurobiol. Dis..

[112] Zhuang X., Zhan B., Jia Y., Li C., Wu N., Zhao M., Chen N., Guo Y., Du Y., Zhang Y. (2022). IL-33 in the basolateral amygdala integrates neuroinflammation into anxiogenic circuits via modulating BDNF expression. Brain Behav. Immun..

[113] O’Loughlin E., Pakan J.M.P., Yilmazer-Hanke D., McDermott K.W. (2017). Acute in utero exposure to lipopolysaccharide induces inflammation in the pre- and postnatal brain and alters the glial cytoarchitecture in the developing amygdala. J. Neuroinflammation.

[114] Jiang X., Liu J., Lin Q., Mao K., Tian F., Jing C., Wang C., Ding L., Pang C. (2017). Proanthocyanidin prevents lipopolysaccharide-induced depressive-like behavior in mice via neuroinflammatory pathway. Brain Res. Bull..

[115] Yang S., Chen X., Xu Y., Hao Y., Meng X. (2020). Effects of metformin on lipopolysaccharide-induced depressive-like behavior in mice and its mechanisms. Neuroreport.

[116] Araki R., Hiraki Y., Nishida S., Inatomi Y., Yabe T. (2016). Gomisin N ameliorates lipopolysaccharide-induced depressive-like behaviors by attenuating inflammation in the hypothalamic paraventricular nucleus and central nucleus of the amygdala in mice. J. Pharmacol. Sci..

[117] Araki R., Hiraki Y., Yabe T. (2014). Genipin attenuates lipopolysaccharide-induced persistent changes of emotional behaviors and neural activation in the hypothalamic paraventricular nucleus and the central amygdala nucleus. Eur. J. Pharmacol..

[118] Hashimoto O., Kuniishi H., Nakatake Y., Yamada M., Wada K., Sekiguchi M. (2020). Early life stress from allergic dermatitis causes depressive-like behaviors in adolescent male mice through neuroinflammatory priming. Brain Behav. Immun..

[119] Zhang J., Lin W., Tang M., Zhao Y., Zhang K., Wang X., Li Y. (2020). Inhibition of JNK ameliorates depressive-like behaviors and reduces the activation of pro-inflammatory cytokines and the phosphorylation of glucocorticoid receptors at serine 246 induced by neuroinflammation. Psychoneuroendocrinology.

[120] Zhang Y.P., Wang H.Y., Zhang C., Liu B.P., Peng Z.L., Li Y.Y., Liu F.M., Song C. (2018). Mifepristone attenuates depression-like changes induced by chronic central administration of interleukin-1beta in rats. Behav. Brain Res..

[121] Chen J., Song Y., Yang J., Zhang Y., Zhao P., Zhu X.J., Su H.C. (2013). The contribution of TNF-alpha in the amygdala to anxiety in mice with persistent inflammatory pain. Neurosci. Lett..

[122] Luo L., Sun T., Yang L., Liu A., Liu Q.Q., Tian Q.Q., Wang Y., Zhao M.G., Yang Q. (2020). Scopoletin ameliorates anxiety-like behaviors in complete Freund’s adjuvant-induced mouse model. Mol. Brain.

[123] Almeida-Suhett C.P., Graham A., Chen Y., Deuster P. (2017). Behavioral changes in male mice fed a high-fat diet are associated with IL-1beta expression in specific brain regions. Physiol. Behav..

[124] Akter S., Uddin K.R., Sasaki H., Shibata S. (2020). Gamma oryzanol alleviates high-fat diet-induced anxiety-like behaviors through downregulation of dopamine and inflammation in the amygdala of mice. Front. Pharmacol..

[125] Sasaki A., de Vega W.C., St-Cyr S., Pan P., McGowan P.O. (2013). Perinatal high fat diet alters glucocorticoid signaling and anxiety behavior in adulthood. Neuroscience.

[126] Sasaki A., de Vega W., Sivanathan S., St-Cyr S., McGowan P.O. (2014). Maternal high-fat diet alters anxiety behavior and glucocorticoid signaling in adolescent offspring. Neuroscience.

[127] Yue J., Wang X.S., Guo Y.Y., Zheng K.Y., Liu H.Y., Hu L.N., Zhao M.G., Liu S.B. (2018). Anxiolytic effect of CPEB1 knockdown on the amygdala of a mouse model of inflammatory pain. Brain Res. Bull..

[128] Wang X.S., Guan S.Y., Liu A., Yue J., Hu L.N., Zhang K., Yang L.K., Lu L., Tian Z., Zhao M.G. (2019). Anxiolytic effects of Formononetin in an inflammatory pain mouse model. Mol. Brain.

[129] Yang L., Wang M., Guo Y.Y., Sun T., Li Y.J., Yang Q., Zhang K., Liu S.B., Zhao M.G., Wu Y.M. (2016). Systemic inflammation induces anxiety disorder through CXCL12/CXCR4 pathway. Brain Behav. Immun..

[130] Doenni V.M., Song C.M., Hill M.N., Pittman Q.J. (2017). Early-life inflammation with LPS delays fear extinction in adult rodents. Brain Behav. Immun..

[131] Xu Y., Day T.A., Buller K.M. (1999). The central amygdala modulates hypothalamic-pituitary-adrenal axis responses to systemic interleukin-1beta administration. Neuroscience.

[132] Weidenfeld J., Itzik A., Goshen I., Yirmiya R., Ben-Hur T. (2005). Role of the central amygdala in modulating the pituitary-adrenocortical and clinical responses in experimental herpes simplex virus-1 encephalitis. Neuroendocrinology.

[133] Sawchenko P.E., Li H.Y., Ericsson A. (2000). Circuits and mechanisms governing hypothalamic responses to stress: A tale of two paradigms. Prog. Brain Res..

[134] Thrivikraman K.V., Su Y., Plotsky P.M. (1997). Patterns of Fos-Immunoreactivity in the CNS Induced by Repeated Hemorrhage in Conscious Rats: Correlations with Pituitary-Adrenal Axis Activity. Stress.

[135] Dayas C.V., Buller K.M., Day T.A. (1999). Neuroendocrine responses to an emotional stressor: Evidence for involvement of the medial but not the central amygdala. Eur. J. Neurosci..

[136] Prewitt C.M., Herman J.P. (1997). Hypothalamo-Pituitary-Adrenocortical Regulation Following Lesions of the Central Nucleus of the Amygdala. Stress.

[137] Frenois F., Moreau M., O’Connor J., Lawson M., Micon C., Lestage J., Kelley K.W., Dantzer R., Castanon N. (2007). Lipopolysaccharide induces delayed FosB/DeltaFosB immunostaining within the mouse extended amygdala, hippocampus and hypothalamus, that parallel the expression of depressive-like behavior. Psychoneuroendocrinology.

[138] Marvel F.A., Chen C.C., Badr N., Gaykema R.P., Goehler L.E. (2004). Reversible inactivation of the dorsal vagal complex blocks lipopolysaccharide-induced social withdrawal and c-Fos expression in central autonomic nuclei. Brain Behav. Immun..

[139] Miller A.H. (2009). Norman cousins lecture. mechanisms of cytokine-induced behavioral changes: Psychoneuroimmunology at the translational interface. Brain Behav. Immun..

[140] Modoux M., Rolhion N., Mani S., Sokol H. (2021). Tryptophan metabolism as a pharmacological target. Trends Pharmacol. Sci..

[141] O’Mahony S.M., Clarke G., Borre Y.E., Dinan T.G., Cryan J.F. (2015). Serotonin, tryptophan metabolism and the brain-gut-microbiome axis. Behav. Brain Res..

[142] Robinson C.M., Hale P.T., Carlin J.M. (2005). The role of IFN-gamma and TNF-alpha-responsive regulatory elements in the synergistic induction of indoleamine dioxygenase. J. Interferon Cytokine Res..

[143] O’Connor J.C., Andre C., Wang Y., Lawson M.A., Szegedi S.S., Lestage J., Castanon N., Kelley K.W., Dantzer R. (2009). Interferon-gamma and tumor necrosis factor-alpha mediate the upregulation of indoleamine 2,3-dioxygenase and the induction of depressive-like behavior in mice in response to bacillus Calmette-Guerin. J. Neurosci..

[144] O’Connor J.C., Lawson M.A., Andre C., Moreau M., Lestage J., Castanon N., Kelley K.W., Dantzer R. (2009). Lipopolysaccharide-induced depressive-like behavior is mediated by indoleamine 2,3-dioxygenase activation in mice. Mol. Psychiatry.

[145] Zhu C.B., Blakely R.D., Hewlett W.A. (2006). The proinflammatory cytokines interleukin-1beta and tumor necrosis factor-alpha activate serotonin transporters. Neuropsychopharmacology.

[146] Moron J.A., Zakharova I., Ferrer J.V., Merrill G.A., Hope B., Lafer E.M., Lin Z.C., Wang J.B., Javitch J.A., Galli A. (2003). Mitogen-activated protein kinase regulates dopamine transporter surface expression and dopamine transport capacity. J. Neurosci..

[147] Duval E.R., Javanbakht A., Liberzon I. (2015). Neural circuits in anxiety and stress disorders: A focused review. Ther. Clin. Risk Manag..

[148] Tovote P., Fadok J.P., Luthi A. (2015). Neuronal circuits for fear and anxiety. Nat. Rev. Neurosci..

[149] Kim M.J., Loucks R.A., Palmer A.L., Brown A.C., Solomon K.M., Marchante A.N., Whalen P.J. (2011). The structural and functional connectivity of the amygdala: From normal emotion to pathological anxiety. Behav. Brain Res..

[150] Muscatell K.A., Dedovic K., Slavich G.M., Jarcho M.R., Breen E.C., Bower J.E., Irwin M.R., Eisenberger N.I. (2015). Greater amygdala activity and dorsomedial prefrontal-amygdala coupling are associated with enhanced inflammatory responses to stress. Brain Behav. Immun..

[151] Dehdar K., Mahdidoust S., Salimi M., Gholami-Mahtaj L., Nazari M., Mohammadi S., Dehghan S., Jamaati H., Khosrowabadi R., Nasiraei-Moghaddam A. (2019). Allergen-induced anxiety-like behavior is associated with disruption of medial prefrontal cortex—Amygdala circuit. Sci. Rep..

[152] Gholami-Mahtaj L., Mooziri M., Dehdar K., Abdolsamadi M., Salimi M., Raoufy M.R. (2022). ACC-BLA functional connectivity disruption in allergic inflammation is associated with anxiety. Sci. Rep..

[153] Zhang W.H., Liu W.Z., He Y., You W.J., Zhang J.Y., Xu H., Tian X.L., Li B.M., Mei L., Holmes A. (2019). Chronic stress causes projection-specific adaptation of amygdala neurons via small-conductance calcium-activated potassium channel downregulation. Biol. Psychiatry.

[154] Ma H., Li C., Wang J., Zhang X., Li M., Zhang R., Huang Z., Zhang Y. (2021). Amygdala-hippocampal innervation modulates stress-induced depressive-like behaviors through AMPA receptors. Proc. Natl. Acad. Sci. USA.

[155] Bourhy L., Mazeraud A., Costa L.H.A., Levy J., Rei D., Hecquet E., Gabanyi I., Bozza F.A., Chretien F., Lledo P.M. (2022). Silencing of amygdala circuits during sepsis prevents the development of anxiety-related behaviours. Brain.

[156] Zhou W., Jin Y., Meng Q., Zhu X., Bai T., Tian Y., Mao Y., Wang L., Xie W., Zhong H. (2019). A neural circuit for comorbid depressive symptoms in chronic pain. Nat Neurosci.

[157] Yuan T., Orock A., Greenwood-Van Meerveld B. (2021). Amygdala microglia modify neuronal plasticity via complement C1q/C3-CR3 signaling and contribute to visceral pain in a rat model. Am. J. Physiol. Gastrointest. Liver Physiol..

[158] Zhang X., Lei B., Yuan Y., Zhang L., Hu L., Jin S., Kang B., Liao X., Sun W., Xu F. (2020). Brain control of humoral immune responses amenable to behavioural modulation. Nature.

